# Face Scanning in Autism Spectrum Disorder and Attention Deficit/Hyperactivity Disorder: Human Versus Dog Face Scanning

**DOI:** 10.3389/fpsyt.2015.00150

**Published:** 2015-10-23

**Authors:** Mauro Muszkat, Claudia Berlim de Mello, Patricia de Oliveira Lima Muñoz, Tania Kiehl Lucci, Vinicius Frayze David, José de Oliveira Siqueira, Emma Otta

**Affiliations:** ^1^Departamento de Psicobiologia, Universidade Federal de São Paulo, São Paulo, Brazil; ^2^Programa de Pós Graduação em Educação e Saúde, Universidade Federal de São Paulo, São Paulo, Brazil; ^3^Departamento de Psicologia Experimental, Instituto de Psicologia, Universidade de São Paulo, São Paulo, Brazil

**Keywords:** face scanning, ASD, ADHD, eye tracking, neurodevelopmental disorders

## Abstract

This study used eye tracking to explore attention allocation to human and dog faces in children and adolescents with autism spectrum disorder (ASD), attention deficit/hyperactivity disorder (ADHD), and typical development (TD). Significant differences were found among the three groups. TD participants looked longer at the eyes than ASD and ADHD ones, irrespective of the faces presented. In spite of this difference, groups were similar in that they looked more to the eyes than to the mouth areas of interest. The ADHD group gazed longer at the mouth region than the other groups. Furthermore, groups were also similar in that they looked more to the dog than to the human faces. The eye-tracking technology proved to be useful for behavioral investigation in different neurodevelopmental disorders.

## Introduction

The study of behavioral and neurophysiological patterns related to visual attention is a promising research field to understand the modulation of attentional performance at different ages and in various neurodevelopmental and neuropsychiatric disorders (1, 2).

Although difficulties in social interaction, empathy, facial expression, recognition, and emotional exchange are core symptoms of autism spectrum disorder (ASD), it has been reported that people with attention deficit/hyperactivity disorder (ADHD) may also have impairment in social cognition and mood regulation, which may lead to high levels of peer rejection (3–6). Social cognition impairments observed in children with ADHD usually involve difficulties in understanding emotional cues especially in negative contexts, such as anger, sadness and disgust, inadequate emotional reaction to emotional perception, and poor ability to inhibit and regulate emotional and behavioral responses (7). At the same time, changes in selective and sustained attention, one of the most consistent conditions in defining the neuropsychological profile of children with ADHD, are also observed in children with ASD (8, 9).

The current DSM-5 (10) does not exclude ASD in the delimitation of ADHD diagnostic criteria, given the frequent association and comorbidity between the two disorders. The existence of shared biological processes in these two neurodevelopmental conditions has been confirmed in epigenetic (11) and neuroimaging studies (12). Behavioral and neurophysiological measures can help to elucidate these disorders. The identification of objective performance measures as endophenotypic markers underlying the common clinical manifestations may be helpful to improve the differential diagnosis.

Attentional modulation occurs in ADHD at different levels, influencing selective attention guided both to external information and to endogenous processes, linked to executive control and emotional self-regulation (3). In this sense, research on motivated attention can contribute to a better understanding of symptom overlap with ASD. Eye movements are privileged pathways for obtaining knowledge about developmental abnormalities, opening new windows into the working of the mind (13). Eye-tracking technology allows a non-intrusive continuous measurement of attention to different types of visual stimuli and can be coupled to other recording devices to get a more complete picture of the physiological events that occur in the brain during information processing and improve our understanding of the neurophysiological and behavioral bases of ADHD and ASD. Although eye tracking has long been used to investigate the gaze patterns of normal adults, only recently it has been employed to study individuals with neurodevelopmental disorders. For instance, using eye-tracking technique, Riby and Hancock (14) compared how individuals with autism (ASD) and Williams’ syndrome (WS) investigated pictures of social scenes. Those with ASD spent less time than is typical viewing people and faces, whereas those with WS showed exaggerated fixations toward faces, and particularly toward the eyes. This study illustrates how the eye-tracking technique can be used to provide markers for atypical sociability and visual attention in neurodevelopmental disorders. Pelphrey et al. (15) reported anomalous face processing among children and adults with autism, with a greater proportion of their inspection time viewing non-feature areas of the faces and a smaller percentage of time examining core features, such as the nose, mouth, and, in particular, the eyes in comparison with control participants. Tottenham et al. (16) also found that individuals with ASD showed fewer gazes toward the eye region and that this behavioral pattern was accompanied by greater amygdala activation to neutral faces in comparison with controls. Dalton et al. (17) extended the use of eye tracking to the relatives of individuals with autism and found that the unaffected siblings’ gaze fixations and brain activation patterns during a face processing task were similar to that of the autism group compared with a matched control group.

### Aims of the Present Paper

The present study aimed to analyze face scanning in two neurodevelopmental disorders. Using eye-tracking techniques, we compared how children and adolescents with ASD, ADHD, and typical development (TD) scanned faces. In contrast with previous studies which have been conducted with high-functioning ASD individuals [e.g., Ref. (15, 18, 19)], our sample included only low-functioning ASD individuals, considering that almost nothing is known about the low-functioning end of the autism spectrum.

In our study, we compared the scanning of human and dog faces by children and adolescents with ASD, ADHD, and typical development (TD). There are well-documented benefits of human–animal interactions for humans of different ages, with and without special mental health conditions with respect to: social attention, social behavior, interpersonal interactions, and mood; stress-related parameters, such as cortisol, heart rate, and blood pressure; self-reported fear and anxiety; and mental and physical health (20). There are reports of changes in prosocial behaviors among autistic children associated to the arrival of a pet in the family (21). It has been proposed that the activation of the oxytocin system plays a key role in these beneficial psychological and psychophysiological effects of human–animal interactions. Oxytocin may be released via eye contact in response to a single meeting with a dog (22). When given the choice to interact with a person, a dog, or an object, children with autism interacted most frequently and for the longest amount of time with the dog (23). Dogs may communicate their intentions in a way more readily understandable to people with autism. Temple Grandin (24–26), a high-functioning autistic woman, who became a renowned professor at Colorado State University, reported that autistic people are closer to animals than normal people are and that looking in the eyes of people is aversive for them.

Photo prints used in a preliminary study, which yielded less gaze aversion to dog than to human faces among ASD children (27), were converted into digital pictures. The results of our pilot study are compatible with the report of Temple Grandin suggesting that the gaze of a dog may trigger less emotional activation than a human gaze.

Our intent was to increase the accuracy of our measurements with the use of eye-tracking technology, extending the investigation to samples of ADHD and typically developing children. The results of this type of research could help to obtain behavioral data useful to the understanding of the differential and shared neuropsychological endophenotype underlying the processing of social–emotional cues in ASD and ADHD conditions.

## Materials and Methods

### Participants

The sample (*N* = 45) consisted of children and adolescents, 15 with typical development (TD) controls (mean 9.5 years, SD = 3.8, 9 girls and 6 boys), 15 with ASD (mean 11.6 years, SD = 2.7, 2 girls and 13 boys), and 15 with ADHD (mean 9.4 years, SD = 2.3, 3 girls and 12 boys). There was a male predominance in both clinical groups in line with the known prevalence of these disorders. The participants were evaluated in a Children’s Interdisciplinary Neuropsychological Care Center in São Paulo, Brazil, by neuropsychologists, pediatric neurologists, and child psychiatrists using DSM-V criteria. The ADHD group was composed of individuals with IQs in the normal range (scores above 85 on WISC-IV), matched to the control TD group. None of ADHD children were taking psycho-stimulant medication at the time of assessment. The Child Behavior Checklist (CBCL) was used as a screen for psychiatric comorbidity in both controls and ADHD groups. The ASD group was composed of low-functioning individuals (IQ below 70) and with scores between 30 and 50 on the childhood autism rating scale – CARS (28).

### Visual Stimuli

Presented stimuli consisted of color photographs of forward facing male and female human faces and dog faces with neutral expression and of neutral control stimuli (clouds and plant), taken from the Karolinska Directed Emotional Faces set (KDEF) (29) and from the International Affective Pictures System (IAPS) (30, 31).

### Data Capture Procedures

**Figure 1:**
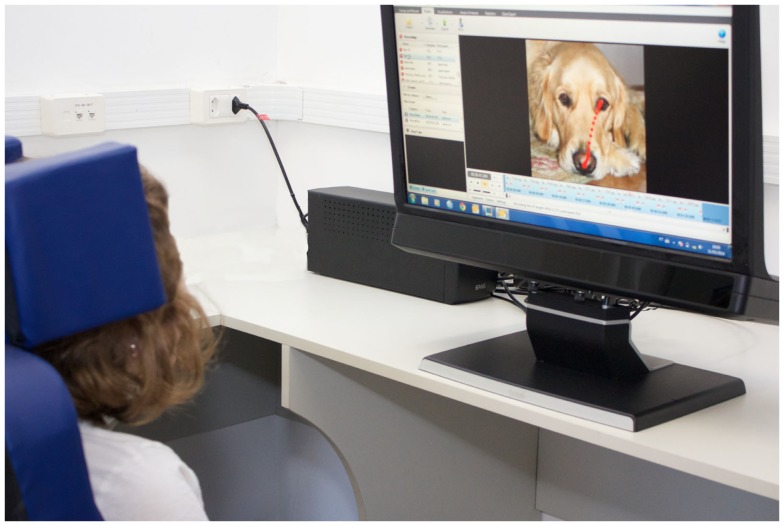
**Illustration of the experimental situation using a video-based eye-tracking system in our laboratory**. Using an eye tracker, we can monitor where the child is looking (photograph of Sarah Kuwano Molinari Salotti).

The eye movements of the participants were recorded with an infrared-based eye-tracking system (Tobii TX 300), integrated with a 23″ TFT monitor (with screen resolution 1920 × 1080 pixel). This equipment used a 300-Hz tracking frequency to collect information about the location and duration of the participant’s gaze fixations on stimuli displayed on the monitor screen (Figure 1). Target static images were presented individually on the monitor for 5 s, separated by a screen showing two rolling dices for 2 s, in order to reduce the effect of environmental distractions, keeping participants’ attention on the monitor screen. The order in which the target images were viewed was randomized across participants.

Tests were conducted in three quiet rooms, with similar characteristics, with lights kept at a constant illumination^1^. The eye-tracking equipment is portable and was moved to each testing location. Participants were tested individually seating approximately 60 cm from the screen in a room with illuminance level of 300 lux in order to guarantee best gaze accuracy (0.4°) and precision (0.07°) as described in the manual of Tobii Tx-300. After a comfortable position was achieved, we asked each child to follow a red ball bouncing around the screen in order to obtain a five-point calibration. The study was conducted only with participants whose calibration was successfully achieved as attested by the Tobii Tx-300. Two research assistants were present during data collection, on each side of the participant, one controlling the computer and the other dealing with logistical issues, but they did not interfere with viewing behavior.

### Ethics

The study was approved by the Human Research Ethics Committee at the Institute of Psychology, University of São Paulo, Brazil. Informed consent was obtained from parents.

### Eye-Tracking Data

Two areas of interest (AOIs) of equal size were selected on each stimulus image to determine gaze location: eyes region and mouth region. Two measures were taken: the number of fixations in each AOI and the total fixation time. Fixations were defined as a gaze of at least 200 ms duration within a 50-pixel radius, as recommended by Tobii manual^2^ for static pictures. The ClearView Fixation Filter (Tobii TX-300) was used for eye movement classifications. Statistical analyses of the eye-tracking data of the three groups were performed with a linear mixed model (general linear mixed model – GLMM). This method was used for analyzing the dependent variables total duration and number of fixations. The independent variables were group (TD, ASD, ADHD), type of facial stimulus (dog, man, woman), and AOI (mouth and eyes). The control IVs were age and sex. The first test made by this method in both analyses was the omnibus test of the existence of a significant effect, adopting the 0.05 significance level. If the omnibus test was significant, the fixed effects to be tested in both analyses were the main effects and the interaction of the second and third orders. In these subsequent analyses, Bonferroni correction was used and the level of significance considered was 0.006. At this second step, significant effects were hierarchically identified from the higher order interactions to the main effects. If significant interaction or main effects were identified, *post hoc* comparisons tests were performed. The software used for analysis was the IBM SPSS Statistics 21.

## Results

### Total Fixation Time

Descriptive statistics summarizing total fixation time as a function of group, type of stimulus, and AOI (Table 1) show that participants spent much of the stimulus presentation time (5 s) with their eyes fixed on the AOIs (around the eyes and the mouth). Average time spent fixating on the eye region ranged from 1.86 to 3.38 s and on the mouth from 0.29 to 1.31 s, regardless of the type of stimulus.

**Table 1:** **Descriptive statistics for total fixation time as a function of group, type of stimulus, and AOI**.

Group	Type of stimulus	AOI	Mean	SD
TD	Dog	Mouth	0.77	0.69
		Eyes	3.16	1.21
	Male	Mouth	0.29	0.34
		Eyes	3.38	1.00
	Female	Mouth	0.68	0.73
		Eyes	2.84	1.23
ASD	Dog	Mouth	0.85	0.97
		Eyes	2.53	1.45
	Male	Mouth	0.29	0.61
		Eyes	1.86	1.35
	Female	Mouth	0.20	0.32
		Eyes	2.51	1.29
ADHD	Dog	Mouth	1.31	0.57
		Eyes	2.74	0.58
	Male	Mouth	0.74	0.78
		Eyes	1.91	0.90
	Female	Mouth	0.83	0.81
		Eyes	2.38	1.44

General linear mixed model analysis revealed that the model had explanatory value at a 5% significance level, indicating a significant type of stimulus main effect (*p* = 0.003) and a significant interaction effect between Group and AOI (*p* < 0.001) (Table 2).

**Table 2:** **Summary of GLMM model examining total fixation time as a function of IVs group, type of facial stimulus, and area of interest with age and sex as control IVs**.

Source	df1	df2	*F*	*p*
Model	19	248	16.606	0.000
Sex	1	248	0.993	0.320
Age	1	248	1.175	0.280
Group	2	248	4.020	0.019
Stimulus	2	248	5.788	0.003
AOI	1	248	265.799	0.000
Group × stimulus	4	248	0.790	0.533
Group × AOI	2	248	8.076	0.000
Stimulus × AOI	2	248	0.190	0.827
Group × stimulus × AOI	4	248	1.609	0.172

With respect to type of stimulus, it is notable that, regardless of other factors, participants spent more time gazing at AOIs of dog images in comparison to human images (Table 3). Pairwise comparison, with sequential SIDAK correction, indicated a significant dog × male difference (*p* = 0.003) and a marginally significant difference dog × female (*p* = 0.055). No difference was found between male and female images (*p* = 0.262).

**Table 3:** **Means, SDs, and confidence intervals of total fixation time as a function of type of stimulus**.

Type of stimulus	Mean	SD	Confidence interval	
			Lower	Upper
Dog	1.922	0.106	1.714	2.130
Male	1.439	0.106	1.229	1.648
Female	1.602	0.106	1.392	1.811

Heatmaps of the most attended areas of animal and human faces illustrate both the similarities and the differences among TD, ASD, and ADHD individuals (Figure 3).

**Figure 2:**
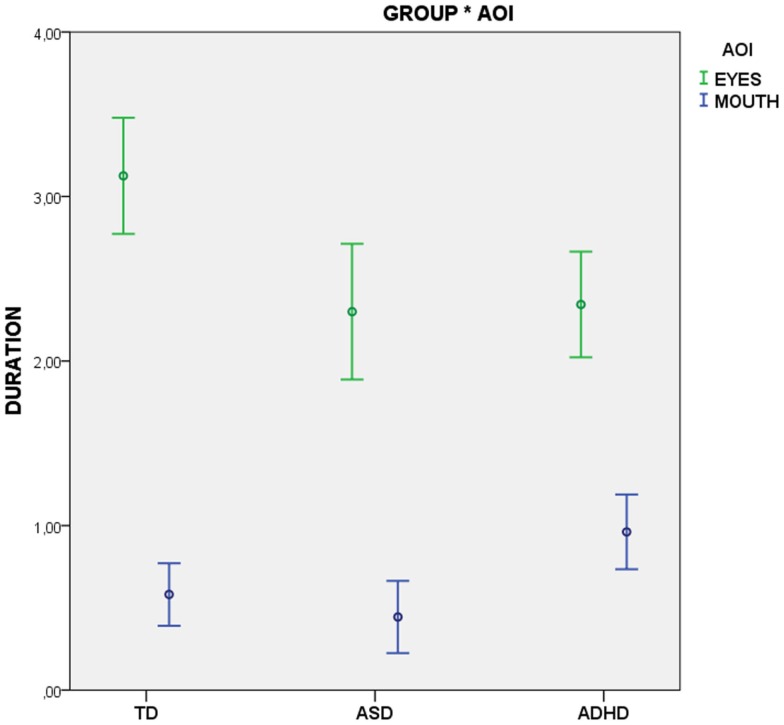
**Total fixation time as a function of type of stimulus**.

The interaction effect represented in Figure 2 shows that, regardless of the stimuli, the typical development group gazed longer at the eyes region in comparison with the other two groups with developmental disorders. Pairwise comparisons with sequential correction SIDAK revealed statistically significant differences of TD versus ASD (*p* = 0.001) and TD versus ADHD (*p* = 0.002). In addition, the ADHD group gazed longer at the mouth region than the other groups. Pairwise comparisons with sequential SIDAK correction indicated significant difference ADHD versus ASD (*p* = 0.025), and a trend toward significance between ADHD and TD (*p* = 0.075).

**Figure 3:**
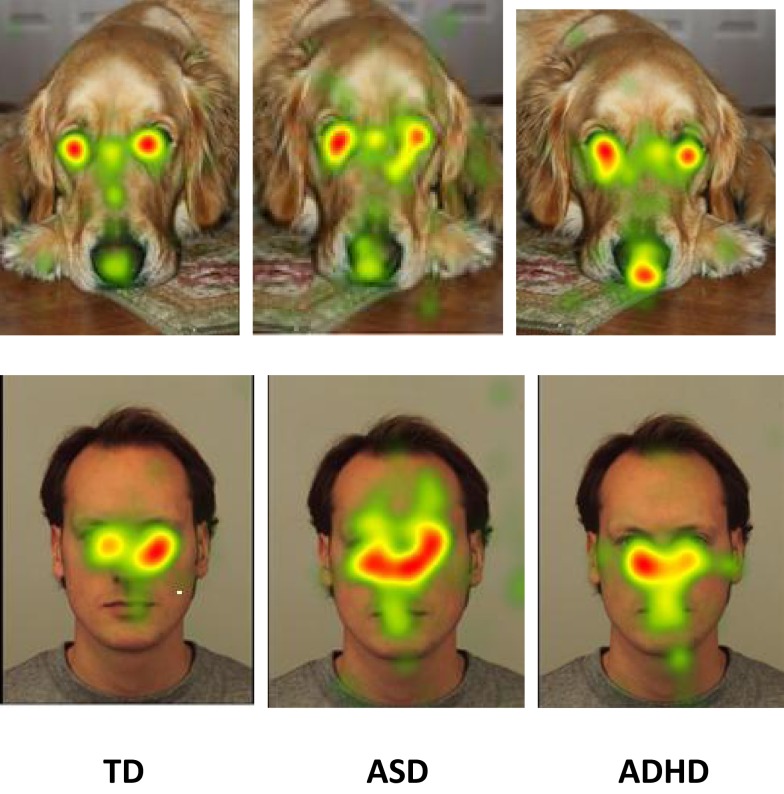
**Heat diagrams illustration of the most attended areas of animal and human faces by TD, ASD, and ADHD individuals**.

## Discussion

In this study, we investigated attentional modulation according to face regions in samples of ADHD, ASD, and typically developing children, by means of eye-tracking procedures. Participants from the ADHD group had considerably higher intellectual and adaptive functioning in comparison to those from ASD group. Nevertheless, they did not differ on time or region of interest of eye tracking, independently of the kind of stimuli. Compared to typically developing controls, the children in the two clinical groups spent less time viewing the eye region than other regions of the face.

This result seems to reinforce previous evidence that difficulties in processing social cues may be shared by both clinical conditions. For instance, using functional neuroimaging techniques, Christakou et al. (11) found a reduced activation in striatal thalamic regions, in superior parietal, and left dorsolateral prefrontal cortex in children with ADHD and ASD in comparison to an age- and IQ-matched control group. Parts of these regions are related to an endogenous processing system known as “Default Network.” The Default network is thought to play an important role in human introspective and adaptive mental activities usually defined as “internal mentation” (32). For instance, it is well known that the theory-of-mind, as other cognitive processes related to socialization skills, is supported by this system (33).

Our results may be summarized in three main findings. The first is that all children looked at dog’s images more than at humans. This apparent greater interest of all participants for dog images suggests a widespread influence of motivational traits in attentional drive in childhood, which can be linked to an evolutionary perspective. A special human interest for other animals can be observed from a very early age (34). “Biophilia Hypothesis” from Wilson (35) proposes that this attraction for nature and life is a result of our evolutionary past.

The second concerns is that the differences in gaze according to face region. All children looked at eyes longer than at the mouth region, but the duration of fixation was lower in ASD and ADHD groups in comparison to TDs. There are several evidences that the processing of facial expressions in ASD children differ than the processing showed by normally developing children. Such differences can be explained by affective or preferences in visual analysis. For instance, an influence of affective valences in the processing of emotions in faces has been reported frequently in ASD children [e.g., Ref. (36)]. A preference for the left visual hemifield in the early stage of visual analysis of faces was observed in typically developing children but not in ASD children when looking to human and also to dog faces (37). In ADHD, on the other hand, such differences are not usually described.

The third main finding is that ADHD children focused on mouth regions more than the other two groups. A possible influence of affective valences in the processing of faces regions may be considered. For instance, Pelc et al. (37) observed, in a face emotion recognition task, that children with ADHD had more difficulties with anger and sadness faces than with other emotions. Problems in emotion recognition were also identified in boys at risk for ADHD (38). Children confounded the emotions of happiness and anger with that of sadness, and spent more time in the eye tracking to identify them. In our study, although we did not investigate specifically responses to emotional valences, the fact that ADHD children have fixed for more time the region of the mouth than the other groups may be related to the importance of the mouth opening for a more precise distinction among positive and negative emotions.

Our findings concerning a possible motivational effect of the interaction with dogs may have clinical implications, for instance, in the planning of alternative behavioral strategies in rehabilitation settings for children with neurodevelopmental disabilities. Theoretical implications include a better understanding of the maturational changes underlying social skill deficits. Finally, we consider that the eye-tracking technology proved to be useful for behavioral investigation even in low-functioning ASD children, and in this way its use in a more natural or ecological assessment setting looks promising.

Some factors may limit generalization of our findings. For instance, we did not include a high-functioning ASD sample for comparison. The most important seems to be the small size and the demographic and clinical heterogeneity of the samples. ADHD were older than ASD children. Participants from ADHD and ASD groups were considerably different in terms of intellectual and adaptive functioning. Nevertheless, they were similar in some aspects of AOI while visually scanning human and dog faces. Other studies will be needed to confirm these findings.

## Conflict of Interest Statement

The authors declare that the research was conducted in the absence of any commercial or financial relationships that could be construed as a potential conflict of interest.

## Footnotes

^1^The data from TD participants were collected in the Didactic Laboratory of Ethology at the Institute of Psychology of the University of São Paulo, the data from ASD participants were collected in the Paulista School of Special Education at São Bernardo do Campo, and the data of participants with ADHD participants were collected in the Paulista Neuropsychology Center/AFIP.

^2^User Manual – Tobii Studio Version 3.2 (http://www.tobii.com).
